# Antimicrobial stewardship principles in the evaluation of empirical carbapenem antibiotics in a private hospital in South Africa

**DOI:** 10.1093/jacamr/dlaf025

**Published:** 2025-03-05

**Authors:** Elmien Bronkhorst, Rose Maboa, Phumzile Skosana

**Affiliations:** School of Pharmacy, Clinical Pharmacy Department, Sefako Makgatho Health Sciences University, Ga-Rankuwa, South Africa; School of Pharmacy, Clinical Pharmacy Department, Sefako Makgatho Health Sciences University, Ga-Rankuwa, South Africa; Life Peglerae Hospital, Rustenburg, South Africa School of Pharmacy, Sefako Makgatho Health Science University, Ga-Rankuwa, South Africa; School of Pharmacy, Clinical Pharmacy Department, Sefako Makgatho Health Sciences University, Ga-Rankuwa, South Africa

## Abstract

**Introduction:**

Antimicrobial resistance, and specifically carbapenem resistance, have developed into a major challenge globally. Because carbapenems are used increasingly as empirical treatment in the presence of rising ESBL infection, the aim of this study was to determine rational prescribing patterns for empirical use of carbapenems. Clinical guidelines are essential in ensuring responsible use in the local context and are one of the most important elements of antibiotic stewardship programmes.

**Methods:**

A retrospective descriptive review of empirical carbapenem use was conducted by reviewing records of participants from an electronic dispensing system and infection prevention pharmacy system. A data collection sheet, which outlines carbapenem utilization evaluation in a large community hospital, was used. Results were analysed descriptively using SPSS (V28) and reported as percentages and frequencies, to provide an overview of the problem.

**Results:**

A total of 450 records were reviewed. Ertapenem was the most frequently prescribed empirical carbapenem. Empirical carbapenem therapy was prescribed mainly for respiratory tract and intra-abdominal infections. Only 15 ESBL-producing organisms were cultured out of the 104 positive cultures. The majority of patients continued with empirical carbapenem therapy despite negative cultures and decreased or normal values of C-reactive protein, procalcitonin and WBC count. Carbapenem prescribing did not comply with guidelines in 70% of the study population, and de-escalation happened in only eight patients.

**Conclusions:**

Antimicrobial stewardship principles were mostly followed, except for correct indication of the antibiotic and de-escalation after culture results. This provided a potential opportunity for intervention to optimize de-escalation to non-carbapenem antibiotics.

## Introduction

Antibiotics constitute the most frequently prescribed medicines in hospitalized patients,^1^ with approximately 71% of patients admitted to the ICU receiving antibiotics.^2^ Antimicrobial stewardship (AMS) is considered an effective strategy to improve the rational use of antimicrobials.^3^ As part of ongoing efforts to conserve effective antibiotics, clinical guidelines for antimicrobial therapy were developed to aid prescribers in rational prescribing of antibiotics.^4^ These principles guide prescribers’ decision-making regarding antibiotic prescribing, including empirical antibiotic therapy.^4^

Clinical guidelines, as developed by the South African Antibiotic Stewardship Programme (SAASP)^4^ or international guidelines like the Sanford guidelines, are essential in ensuring responsible use in the local context and are one of the most important elements of antibiotic stewardship programmes (ASPs).^5^

The rising incidence of infections with ESBL-producing organisms, because of the overuse of β-lactam antibiotics,^6,7^ increased the use of carbapenem antibiotics because of their broad spectrum against Gram-positive and Gram-negative organisms.^8^ Carbapenems are considered the treatment of choice for serious infections due to ESBL-positive Enterobacteriaceae and are considered a last line of defence against Enterobacteriaceae.^9^ This in turn led to the ongoing global problem of carbapenem resistance.^10^ The utilization of carbapenems in the presence of ESBLs as empirical therapy is becoming a widespread phenomenon.^8^ Empirical therapy can be defined as the initial antibiotic regimen selected in the absence of definitive microbiological pathogen identification.^11,12^ Although a delay in initiation of appropriate empirical antibiotic therapy may contribute to increased mortality,^13^ irrational use may result in a significant amount of broad-spectrum antibiotic consumption.^14^

ASPs are implemented to promote strategies to optimize clinical outcomes, minimize costs and prevent side effects of therapy.^15^ These strategic measures include ensuring timely microbiological cultures (before initiation of antimicrobial agents),^16^ as well as re-evaluation of broad-spectrum antibiotics like carbapenems within 48–72 h after initiation.^14,17^ When patients present with a concern for sepsis, the likely source of infection, likely pathogens and how catastrophic outcomes will be when empirical treatment is not appropriate should be considered. The specific antibiotic regimen should be determined using consideration of site-specific diagnostics like blood cultures, identifying the probable causative organism, based on epidemiological and host risk factors.^11^ After culture results are obtained, appropriateness of the empirical antibiotics must be reviewed and opportunities to de-escalate or potentially stop therapy must be considered.^11^ De-escalation is when one or more broad-spectrum antibiotics initiated as empirical therapy are stopped and/or replaced by a narrower-spectrum antibiotic.^18^ This strategy plays a vital role in reducing the emergence of antimicrobial resistance (AMR), side effects and costs.^15^

An increase in the utilization of carbapenems as empirical therapy was observed in the study site, without a significant increase in infections with ESBL-producing organisms. The present study aimed to determine the indications for the utilization of carbapenem antibiotics for indication as empirical therapy, and prevalent bacterial infections in these cases. Furthermore, compliance with appropriate AMS principles according to the SAASP guidelines^4^ and Sanford guidelines were investigated.

## Materials and methods

### Ethics

Ethical clearance was received from the university’s research committee (SMUREC/P/134/2020:PG) and from the hospital group’s Ethical Committee (Ref no: 03102021/6). Data collection commenced after permission was granted by the hospital manager of the study site. Patient consent was not required as data were collected from patient file reviews; however, patient confidentiality was ensured during the process.

### Settings and population

This study was conducted at a 240-bed private hospital in South Africa. Records of all adult patients admitted over a period of 2 years (June 2018—June 2020), irrespective of gender, who were prescribed carbapenems empirically, were eligible for inclusion in the study. Patients older than 18 years who received empirical carbapenem therapy during the study period were purposively identified via the hospital group’s computerized database, and patient history and prescription data were traced using the pharmacy dispensing programme.

The average admission rate to the selected wards was 4078 patients per month. Using the Raosoft^®^ population calculator with a 5% margin of error and 95% CI, a minimum sample size of 384 was required. Data were collected to include all patients for the 2 year period, to yield a final study population of 450.

### Study design and data collection

The study was a quantitative, descriptive study with data collected retrospectively over a period of 2 years. Records were reviewed using a data collection sheet, which was developed with the guidance of a study reported by Janssen and Kinkade,^19^ which outlines carbapenem utilization evaluation in a large community hospital. The tool collected demographic data, diagnosis and medication history of the patients, together with their laboratory and microbiological data. Before reviewing these records, the data collection sheet was pilot tested on the records of 10 patients outside of the study period, and these data were not included in study results. Demographic data, diagnosis and medication history were collected from the billing and dispensing hospital database. Laboratory and microbiological data were collected from the electronic infection control programme used in the hospital.

Prescription results were compared for dose, duration, indication, biomarkers and culture monitoring, as well as de-escalation, against the published guidelines from SAASP and the international guideline from Sanford.

### Statistical analysis

Data were captured onto a Microsoft Excel™ spreadsheet, cleaned and checked for accuracy. It was imported into Statistical Package for Social Sciences (SPSS) version 28, where it was analysed descriptively. The distribution of the data was shown by means of frequencies and percentages, and data were presented as tables and graphs.

## Results

### Patient demographics

A total of 450 patient records that included a carbapenem prescription as empirical therapy were reviewed. There were more male patients (241; 53.6%) than female patients, and the mean age of the study population was 53 (SD ± 15.9) years. Some patients had more than one comorbidity resulting in 627 comorbidities in the 450 patients (1.4 comorbidities per patient), with hypertension (187; 41.7%) the most prevalent of the comorbidities. Comorbidities identified in the study are indicated in Table 1, with the single comorbidities including irritable bowel syndrome, systemic lupus erythematosus and rheumatoid arthritis, amongst others.

**Table 1.** Comorbidities of patients

Comorbidities, *n* (%) (*N* = 450)	
Hypertension	187 (41.6)
Cancer	78 (17.3)
HIV	75 (16.7)
Diabetes mellitus	68 (15.1)
Cardiac diseases	53 (11.7)
Renal failure	37 (8.2)
Lower respiratory tract diseases	54 (12)
Hyperlipidaemia	26 (5.8)
Hypothyroidism	15 (3.3)
Epilepsy	14 (3.1)
Other	13 (2.8)
Penicillin allergy	7 (1.6)

### Prior antibiotic exposure

**Figure 1.**
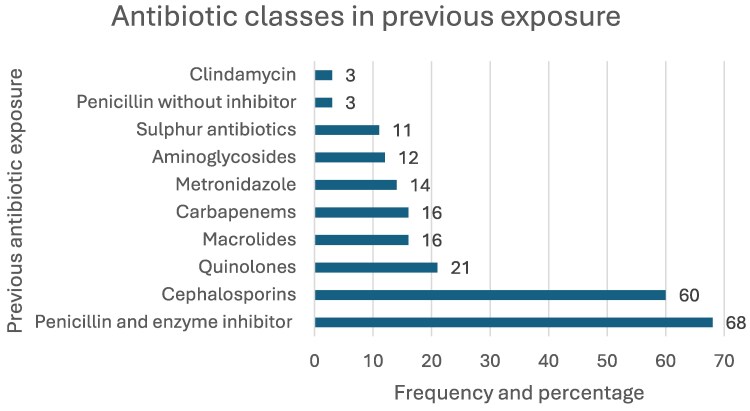
Antibiotic classes used in previous exposure.

Some patients were exposed to antibiotics before the initiation of carbapenems. Almost half (216; 48%) of the patients received a course of antibiotics either during their current hospital stay, or within 3 months prior to admission. Figure 1 shows the common antibiotic classes that were prescribed to patients within the previous 3 months, with penicillin/enzyme-inhibitor combinations (68; 30.3%) and cephalosporins (60; 26.8%) being the top two.

### Admission diagnosis

**Figure 2.**
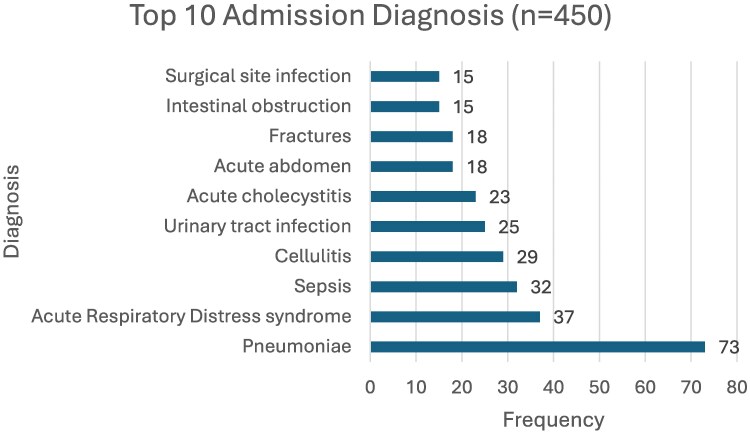
Admission diagnosis.

Admission diagnosis is a consideration in the selection of appropriate empirical antibiotics. Most patients who were admitted to the medical wards (56%) were treated for lower respiratory tract infections (142; 29.7%), specifically pneumonia (73; 16.2%) and acute respiratory distress syndrome (37; 8.2%). Those admitted to the surgical wards (44%) were treated for gastrointestinal or intra-abdominal infections (112; 23.4%). Although not indicated, non-infectious diagnoses like fractures (18; 4%) and intestinal obstruction (15; 3.3%) were also treated with empirical carbapenem therapy. Figure 2 provides an overview of admission diagnosis.

### Frequency of empirical carbapenem use

The majority of empirical carbapenems were prescribed for community-acquired infections (379, 84.2%), within 48 h after admission to the hospital. Ertapenem was the most frequently prescribed (338; 75.1%), especially in the surgical and medical wards, followed by meropenem (93; 20.7%). Most patients (275; 61.1%) were prescribed carbapenem therapy for a duration of 4–7 days. Only one patient received empirical carbapenem therapy for a duration of 21 days.

Several patients (48; 10.6%) received empirical carbapenem therapy because of a secondary diagnosis of suspected infection, after admission for non-infectious primary diagnosis. This includes pleural effusion and sepsis after surgical procedures.

### Biomarkers and microbiological cultures

Biomarkers were monitored in most patients (350; 77.8%) to assess therapy and these included procalcitonin (PCT) in 224 (49.7%), C-reactive protein (CRP) in 336 (74.7%) and WBC count (WCC) in 329 (73.1%) of the patients. Almost half of the patients (166; 47.4%) had increased values for two or more biomarkers at the same time, which indicated infection or inflammation.

Microbiological cultures were obtained in 293 (65.1%) patients, where 272 were obtained before initiation of therapy, and 21 only after initiation of therapy. In some patients, specimens were collected from more than one site in the same patient, giving a total of 439 specimens collected, mostly from blood (162; 36.9%) and urine (130; 36.9%). There were 326 negative culture results from the specimens (74.3%). Table 2 provides an overview of AMS principles followed in the monitoring of correct empirical prescribing and de-escalation of therapy according to the SAASP and Sanford guidelines.

**Table 2.** Microbiology results

Results	*n* (%)
Microbiological cultures (*N* = 450)	
Obtained before initiation of empirical carbapenem therapy	272 (60.4)
No cultures obtained	157 (34.8)
Cultures taken after initiating empirical therapy	21 (4.6)
Specimens obtained before and during the course of empirical carbapenem therapy (*N* = 439)	
Urine	130 (29.6)
Blood	162 (36.9)
Swab (pus, abdominal)	36 (8.2)
Sputum	75 (17)
Aspirates (tracheal, bronchial)	26 (5.9)
Body fluids (CSF, pleural, ascitic)	8 (1.8)
Tissue sample	2 (0.5)
De-escalation of empirical carbapenem therapy where cultures were obtained (*N* = 293; 65.1%)	
Number of patients where therapy was de-escalated	8 (2.7)
Number of patients where therapy was not de-escalated	267 (91.1)
Number of patients where de-escalation was not possible due to organism susceptibility	18 (6.1)

The majority of patients 267 (91.1%) where cultures were taken, continued with empirical carbapenem therapy despite negative cultures and decreased or normal values of CRP, PCT and WCC. In 18 (6.1%) patients, an MDR Gram-negative bacterium was cultured, therefore de-escalation was not applicable.

### Prevalent organisms

During the study period, 104 bacteria were cultured from 293 patients, the most prevalent being: *Escherichia coli*, both ESBL positive and ESBL negative (25; 24.0%); *Staphylococcus aureus*, both MSSA and MRSA (21; 20.2%); and *Enterococcus faecalis* (15; 14.4%). Carbapenems were indicated in all 12 cases of *Pseudomonas aeruginosa*, as resistance to other agents was indicated on the antibiogram. Table 3 represents the organisms that were isolated on the microbiological culture reports obtained from the patient records, and whether the carbapenem prescribed was indicated as first-line treatment for the identified organism, according to the guidelines.

**Table 3.** Correct indication of carbapenem prescribing in relation to cultured organisms

Cultured organisms (104)	Carbapenem therapy appropriate	Carbapenem therapy not appropriate
*Acinetobacter baumannii* (*n* = 4)	4	0
*Enterobacter cloacae* (*n* = 5; 3 ESBL +)	3	2
*E. faecalis* (*n* = 15)	0	15
*E. coli* (*n* = 25; 8 ESBL+)	8	17
*K. pneumoniae* (*n* = 12; 5 ESBL +)	5	7
*M. tuberculosis* (*n* = 7)	0	7
*P. aeruginosa* (*n* = 12*)*	12	0
*S. aureus* (*n* = 21; 4 MRSA)	0	21
Other^a^ (*n* = 20)	9	11

^a^Organisms include *Stenotrophomonas maltophilia*, *Pneumocystis jirovecii*, *Mycoplasma pneumoniae*, CoNS, *Proteus mirabilis*, *Serratia marcescens* and *Raoultella planticola*.

### Risk factors for development of MDR organisms

During this study, risk factors^11^ for the development of MDR infections were evaluated. Patients with immunosuppression (193; 42.9%), including HIV-reactive patients, diabetes mellitus and cancer patients, represented the major risk factors for infections with MDR organisms, and included a large portion of the study population. Advanced age (65 years or older) (139; 30.9%), as well as admission to ICU (98; 21.7%), could further be considered significant risk factors. More than a third of the study population (168; 37.3%) received empirical carbapenem therapy without the presence of a risk factor for the development of MDR organisms.

According to the SAASP and the Sanford guidelines, empirical therapy was not initiated appropriately in most patients enrolled in this study (315; 70%). The strategies that were not complied with, according to the guidelines, were mostly about taking appropriate cultures before initiation of antibiotic therapy (137; 39.6%) and improper de-escalation after identification and susceptibility results for the organisms (421; 93.1%).

## Discussion

The present study was conducted to determine the rational prescribing of carbapenem antibiotics as empirical therapy. Furthermore, the application of AMS principles was investigated and compared with guidelines from SAASP,^4^ as well as Sanford. The aim of the study was to determine whether these principles may be employed to reduce the irrational prescribing of carbapenems.

Comorbidities identified in this study included hypertension, cancer and HIV. Similar findings were reported in other studies in South African hospitals, with cancer, diabetes mellitus and cardiovascular diseases being the most prominent comorbidities amongst patients treated with carbapenem antibiotics.^20,21^ Comorbidities increase the risk for development of sepsis from any type of infection in most patients.^22^

Just less than half of the study population received other types of antibiotics (most prevalent being penicillin/enzyme-inhibitor combinations and cephalosporins) preceding the 3 months prior to initiation of empirical carbapenem therapy. Even without prior exposure to antibiotics, almost half of the population were initiated on carbapenem therapy, without consideration of indicated first-line options and carbapenem-sparing options like piperacillin/tazobactam, cefepime or fluoroquinolones. The reluctance of prescribers to consider non-carbapenem options may be one of the barriers to optimal AMS implementation. Goodlet *et al.*^20^ similarly reported a large portion of their study population with prior exposure to cephalosporins (45.2%), while Wagner et al.^23^ found almost 60% of patients received at least one other antibiotic (mostly cephalosporins and penicillin) within 7 days prior to receiving a carbapenem.^23^ The high incidences of previous antibiotic exposure illustrates the problem of antibiotic overprescribing, increasing antibiotic resistance.

The most prevalent admission diagnoses found were lower respiratory tract infections and gastrointestinal or intra-abdominal infections, which were similar in a study by Entezari-Maleki *et al.*^24^ Secondary diagnosis of sepsis after initial admission for non-infectious reasons included pleural effusion, which could be a symptom of hospital-acquired pneumonia, requiring medical treatment. In their study, Goodlet *et al.*^20^ agreed that 73% of patients received treatment for medical indications at the time of empirical carbapenem therapy,^20^ while Grau *et al.*^10^ reported that 53% of their study population received treatment for hospital-acquired infections.^10^ The high rates of nosocomial infections may highlight the fact that insufficient infection prevention measures, which are also AMS principles, were not followed.

Ertapenem was the most frequently prescribed carbapenem in this study, followed by meropenem. Grau *et al.*,^10^ however, reported meropenem as the most frequently prescribed empirical carbapenem, followed by ertapenem.^10^ Another study by Perron *et al.*^25^ showed that imipenem was the most frequently prescribed carbapenem in a university hospital in France.^25^ However, in the USA, doripenem was the most frequently prescribed carbapenem.^20^ The choice of specific carbapenem is dependent on several factors, like the specific spectrum of activity, local resistance patterns, safety and side effects, and cost and availability. In this study, ertapenem was the choice of antibiotic mostly for community-acquired infections, which is contradictory to guidelines and antibiotics considered should rather include other first-line antibiotics.

Only around a third of prescriptions were compliant with SAASP and Sanford guidelines as per indication for empirical carbapenem therapy. Contrary to this finding, Van Hollebeke *et al.*^26^ reported 74% of patients received empirical carbapenem treatment in line with guidelines.^26^ This contradiction may be attributed to the increase in Gram-negative bacteria observed in South Africa.^27^ At the study facility, however, few ESBL-positive infections were identified, reducing the risk in the facility. Dosing of empirical carbapenem therapy was in line with the recommendations from guidelines in 87% of the study population, in line with Goodlet *et al.*,^19^ who found the majority of their population (77%) received appropriate doses of empirical carbapenems.^20^

The most common indications for prescribing empirical carbapenem therapy were found to be lower respiratory tract and gastrointestinal or intra-abdominal infection. Several studies had similar findings, where the most common indications for the use of empirical therapy were respiratory, intra-abdominal and bloodstream infections.^23,26^ Ertapenem was the choice of empirical antibiotic for the treatment of intra-abdominal infections and second for lower respiratory tract infections, while meropenem was preferred for treatment of lower respiratory tract infections.^26^

Most patients (61%) were on carbapenem therapy for a duration of 4–7 days, in line with Goodlet *et al.*,^20^ with a mean duration for empirical carbapenem therapy of 5.7 days.^20^ In comparison, Wagner *et al*.^23^ found a longer duration of treatment extending to 11 days.^23^

As found in several infectious disease studies, biomarkers like CRP, PCT and WCC were monitored in most patients.^28–30^ Culture samples should be collected from all sites suspected to be the source of infection.^31^ In the current study, the most common specimens required for cultures were urine, blood and sputum samples. However, more than 75% of the requested cultures yielded no growth. AMS measures may be implemented to ensure timely and correct specimen collection, which may increase growth yield of the specimens. Entezari-Maleki *et al.*^24^ found a low growth rate of 24% positive results.^24^ Other studies reported better outcomes with around 50% of specimens yielding a positive growth.^10^ Comparable to other studies, the most frequent Gram-negative organisms cultured in the present study were *Klebsiella pneumoniae*, *E. coli* and *P. aeruginosa*.^10,23^ Amongst these organisms, 15 isolates were ESBL positive, which is not comparable to the high prevalence of ESBL-positive organisms reported by Fourie *et al.*^32^ and Karaiskos and Giamarellou.^33^ High ESBL-positive rates can thus not be the reason for increased utilization of carbapenems for both empirical and directed therapy.^31,32^

Despite efforts made by AMS teams, de-escalation remains suboptimal, especially in ICU and surgery wards.^34^ Amongst the patients who had negative cultures in the current study, the therapy of only eight patients was de-escalated. Furthermore, prescribers were reluctant to de-escalate patients’ therapy even in cases where the cultured organism was inherently resistant to a carbapenem (e.g. *Mycobacterium tuberculosis* and MRSA). The researcher found that even in cases where a narrow-spectrum agent was the first-line option for the cultured organism, de-escalation was refused. In other studies it was found that surgeons are often resistant to agreeing to the recommendations to de-escalate.^35,36^ Reasons stated for this reluctance to de-escalate included the high mortality rate associated with intra-abdominal infections, and the more prevalent MDR organisms, as well as the advanced age of the specific population. A study to evaluate the safety and efficacy of de-escalation in patients on carbapenems reflected that de-escalation is a safe strategy that does not compromise the prognosis of severely ill hospitalized patients.^15^ To support this, studies showed 36% and 40% of patients were successfully de-escalated from empirical carbapenem therapy.^10,20^

Identification of risk factors for development of MDR organisms and infection are measures that can be implemented to devise appropriate preventative and control measures.^37^ Rodriquez-Morales *et al.*^38^ noted that patients of advanced age are generally twice as likely to be colonized with MDR organisms than younger patients. Our study identified immunosuppression and advanced age as major risk factors, which is representative of South Africa’s population, with a high prevalence of HIV and non-communicable diseases such as diabetes mellitus.^38^ Siwakoti *et al.*^39^ stated that despite advances in the ICU setting in recent years, the incidence of MDR bacteria remains higher in the ICU compared with other units.^39^ The present study did not consider the influence of effective infection prevention practices, nor the role of a dedicated AMS pharmacist. This institution has a AMS committee; however, because of personnel shortages, neither the infection prevention specialist nor the pharmacist are active members of the committee, which may change the future incidence of resistant bacteria in the ICU setting.

## Limitations

The nature of the study was retrospective, which limited the opportunity to provide interventions from the pharmacist. Future prospective studies should be performed to better describe the interventional role of the pharmacist in AMS. Furthermore, the study was conducted in only one hospital, thus results can not be generalized to the larger community.

### Conclusions

Empirical carbapenem therapy was directed at a wide range of suspected infections but was found to be non-compliant with SAASP and Sanford guidelines regarding indications for the larger part of the study population. Although microbiological cultures were requested before initiating and administering carbapenem therapy in most patients, prior antibiotic use was not considered. Culture results were negative in most cases with only a few ESBL-positive Gram-negative organisms cultured. This presented an opportunity for using a narrower-spectrum antibiotic. However, de-escalation of therapy proved challenging, as most of the study population continued with empirical carbapenem therapy despite culture results. Consideration of risk factors is essential before initiating empirical antibiotic therapy and can influence the decision to use carbapenem therapy. An interventional study, where the antibiotic stewardship team intervenes in empirical carbapenem treatment could be recommended to reduce unnecessary carbapenem use. This study underscores the ongoing issues with carbapenem antibiotic prescribing and emphasizes the potential role of AMS interventions and guidelines.
